# The impact of smoking and quitting on household expenditure patterns and medical care costs in China

**DOI:** 10.1136/tc.2008.026955

**Published:** 2009-01-21

**Authors:** Y Xin, J Qian, L Xu, S Tang, J Gao, J A Critchley

**Affiliations:** 1 Center for Health Statistics and Information, Ministry of Health, Beijing, PR China; 2 International Health Research Group, Liverpool School of Tropical Medicine, Liverpool L3 5QA, UK; 3 Institute of Health and Society, Newcastle University, Newcastle upon Tyne NE2 4HH, UK

## Abstract

**Background:**

Smoking remains very common in Chinese men, and the economic burden caused by cigarette consumption on smokers and their families may be substantial. Using a large nationally representative household survey, the third National Health Services Survey (NHSS, 2003), we estimated the economic impact of smoking on households.

**Methods:**

Smoking status of all household members (over 15 years) was collected by interview for the NHSS, and households classified into one of seven categories based on their smoking status. Information on household income and expenditure, and use of health services was also obtained. We assessed both the “direct” costs (reducing funds available for spending on other commodities such as food, education, medical care, etc, using a fractional logit model), and “indirect costs” (increasing medical expenditures, using a log-linear model).

**Results:**

Every five packets of cigarettes consumed per capita per month reduces household spending on other commodities, most notably on education (by about 17 yuan per capita per annum) and medical care (11 yuan). The effects are greatest among low-income rural households. Households with quitters spend substantially more on medical care than never-smoking households (64 yuan for households with two or more quitters).

**Conclusions:**

If a household member smokes, there is less money available for commodities such as education and medical care. Medical care expenditure is substantially higher among households with quitters, as ill-health is the main reason for quitting smoking in China. Smoking impoverishes a substantial number of poorer rural households.

Smoking prevalence remains very high among Chinese men. The 2002 national smoking survey estimated an ever-smoking rate of 66% for men and 3.1% for women.^1^ Although Western cigarette brands sold in cities can be more expensive, local brands in rural areas are as cheap as 2–3 yuan per packet (there were approximately 7 yuan to $US1 as of November 2008). An average price of about 3.71 yuan per packet was reported by the Chinese statistical bureau in 2002.^1^ ^2^ Although the cost of smoking increased in real terms from the late 1980s until the late 1990s,^3^ ^4^ China’s GDP has also risen significantly over recent years, and cigarettes may have become more affordable. Nevertheless, approximately 9–11% of rural households live below the poverty line in China (on less than the equivalent of $1 daily),^5^ ^6^ and the prevalence of smoking is higher in rural than in urban parts of China.^7^

The economic burden of smoking on households in China could therefore be substantial.^8^ The direct consumption of tobacco has an opportunity cost, restricting the household budget available to spend on other goods and services. Smoking may also result in “indirect costs” to households, particularly excess medical expenditure for treatment of smoking-related diseases and conditions, as a result of both active smoking and exposure to environmental tobacco smoke (ETS) among other family members.

Few studies have examined the impact of tobacco spending on household budgets in low or middle-income countries,^9^ and even fewer in large, nationally representative samples. Tobacco expenditure may cause substantial harm to poor households with limited incomes for food and basic needs. Analysis of data from a survey of approximately 4500 households in rural China showed that smoking expenses can harm other family members by reducing expenditures on basic needs such as foods, utilities and durable goods.^9^

The main aims of this study were therefore:

1. To examine the effects of tobacco consumption on other household expenditures
2. To estimate the excess medical spending attributable to smoking and exposure to ETS.

## METHOD

### Data source

This study used data derived from the third National Health Services Survey (NHSS) conducted in 2003, a nationally representative survey which covered both urban and rural populations, and collected information on healthcare utilisation and expenditure. The survey adopted multistage stratified cluster random sampling, and the methodology has been published elsewhere.^10^^–^13 The overall sample included 95 counties (districts), 475 townships (streets) and a total of 950 villages (resident committees). The basic sampling unit was the household, and 60 households were randomly sampled in each village or resident committee. Enumeration was undertaken by selected local health workers who undertook face-to-face interviews in the respondent’s home. The survey collected data on demographic and socioeconomic factors, self-reported health status, healthcare utilisation, household incomes and expenditures on a variety of commodities, medical expenditure, and behavioural factors such as smoking and drinking at an individual level. A total of 57 023 households, including 193 689 people, took part. Over 99% of households initially selected agreed to take part, and 99.6% of respondents answered questions on smoking status. In total, 56 916 households answered questions on both smoking status and household expenditures, and were therefore included in our study.

### Smoking status of households

Those who had smoked at least five packs of cigarettes during their lifetime (100 cigarettes in total), and reported smoking at the time of the survey are defined as “smokers” in this study. Former smokers are defined as those who had smoked at least 100 cigarettes over their lifetime, but had stopped by the time of the survey. All current smokers were asked how many cigarettes they consumed daily (coded as the exact number). All members of the household over 15 were interviewed; we can therefore estimate total household tobacco consumption.

All households were divided into seven mutually exclusive categories according to the smoking status of family members (see table 1). Never-smoking households are those with no current or former smokers. Households with at least one current smoker (and no former smokers) were classified based on the total household consumption of cigarettes (in percentiles). Those in the 0–25 percentile of tobacco consumption were defined as low-tobacco consumption households. Likewise, those in the 25–75 percentile or 75–100 percentiles were categorised as moderate or high-tobacco consumption households. Households with former smokers only were categorised according to the number of former smokers (one, two or more). Households with both current smokers and quitters were classified as “mixed” consumption (table 1), and not included in the low, medium or high categories.

**Table 1:** The number and percentage of households by smoking status, National Health Services Survey (NHSS) survey

Household groupings	National	Urban	Rural
	No (%)	No (%)	No (%)
Never-smoking households* (NS)	21 588 (37.9)	7284 (43.4)	14 304 (35.6)
Low tobacco consumption (Low)	10 602 (18.8)	3499 (20.9)	7205 (18.0)
Medium tobacco consumption (Medium)	14 447 (26.6)	3638 (21.7)	11 504 (28.7)
High tobacco consumption (High)	7702 (12.1)	1440 (8.6)	5465 (13.6)
One former smoker† (1 quit)	1049 (1.8)	341 (2.0)	708 (1.8)
Two or more former smokers† (2+ quit)	934 (1.6)	389 (2.3)	545 (1.4)
Mixed (smokers and quitters)‡ (Mixed)	594 (1.0)	192 (1.1)	402 (1.0)
Total	56 916 (100)	16 783 (100)	40 133 (100)

*No current or former smokers in the household, NS (household of never-smokers).

†No current smokers but there are one (1 quit) or at least two former smokers (2+ quit) who have quit smoking in the household.

‡At least one current smoker, and also at least one former smoker who has quit in the household.

Low, low tobacco consumption (0–25% of distribution of household tobacco consumption); Medium (25–75%); and High tobacco consumption (75–100%).

The categories are mutually exclusive—ie, “mixed” households with smokers and quitters are not also counted as low, medium or high consumption households.

Exposure to ETS is a serious problem in China.^1^ ETS occurs in the workplace and other public places, and in the home. We could only assess exposure to ETS at home, as the NHSS requested only limited information on exposure elsewhere. The survey asked whether there were any restrictions on smoking in the household so, conservatively, we defined ETS exposure as occurring among households with at least one current smoker, and no smoke-free policies in the home.

## ANALYSIS

### Direct costs of smoking—household consumption and expenditure patterns

Initially, we calculated the percentage of total household expenditures spent on seven distinct expenditure categories (food, clothing, transportation, housing, education, medical care, and others). These seven categories are exhaustive and mutually exclusive. Table 2 gives the crude mean expenditure for these seven categories, by household smoking status. Expenditure on tobacco was not directly collected by the survey, so is included in the “Others” category.

**Table 2:** Descriptive statistics on household expenditure (yuan and %) by household tobacco consumption

	NS	Low	Medium	High	1 Quit	2+ Quit	Mixed
**Average household consumption by smoking status**							
Number of households	21 588	10 602	14 447	7702	1049	934	594
Number of individuals	66 818	37 072	52 753	27 877	3713	2452	2686
Expenditure by category (yuan)*							
Food	1152	925	952	1179	1183	1527	1100
Clothing	279	228	242	271	244	286	239
Transportation	228	175	194	256	217	252	228
Housing	265	200	204	269	268	353	240
Education	390	291	327	342	392	365	237
Medical care	327	221	240	328	459	786	383
Others	216	173	196	274	190	287	184
**Percentage of total household expenditure for each category**							
Food	40.3	41.8	40.4	40.4	40.1	39.6	42.1
Clothing	9.8	10.3	10.3	9.3	8.3	7.4	9.1
Transportation	8.0	7.9	8.2	8.8	7.3	6.5	8.7
Housing	9.3	9.0	8.7	9.2	9.1	9.1	9.2
Education	13.6	13.1	13.9	11.7	13.3	9.5	9.1
Medical care	11.4	10.0	10.2	11.3	15.5	20.4	14.7
Others	7.6	7.8	8.3	9.4	6.4	7.4	7.0

*There are approximately $68, or €8.9 to 1 yuan (data as of November 2008). NS, household of never-smokers; Low, low tobacco consumption (0–25% of distribution of household tobacco consumption); Medium (25–75%); High tobacco consumption (75–100%); 1 Quit, household with one quitter only and no current smokers; 2+ Quit, household with two or more quitters and no current smokers; Mixed, household with both current smokers and quitters.

Our aim was to estimate how household spending on tobacco affects spending on other goods and services, after controlling for other household characteristics. Because a category for expenditure on smoking was not included in the NHSS, we cannot estimate smoking costs directly. The amount of tobacco consumed by the households was therefore the predictor variable (per five packets, or 100 cigarettes per month). Our outcome variable is the percentage of total household expenditure allocated to each category; we therefore used the fractional logit model to predict the effect of the number of cigarettes smoked on household expenditure patterns after controlling for other factors.^9^ ^14^^–^16 Using the fractional logit model, the predicted values of these percentages will be bounded by the unit interval [0, 1].

Specifically confounders considered a priori and included were household head’s age, gender, marital status, level of education, occupation, insurance and self-reported health status; household location (urban or rural area), household income, number of household members, household members under 15 years or over 65 years and the number of former smokers (see appendix 1).

We ran the following model on each of the seven household expenditure categories to predict the household tobacco consumption effects:


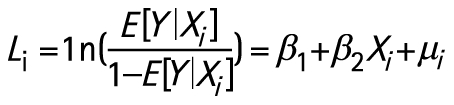



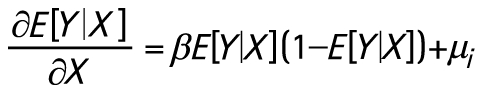


where *L* represents the logit function, Y represents the percentage of expenditures spent on each of our seven categories, X represents tobacco consumption, as well as our control variables and *μ_i_* is an error term.

After construction of the prediction model for each expenditure category, a “recycling” prediction method was used to predict the percentage for each category based on the estimated coefficients.^17^

### Increase in medical expenditures associated with smoking and ETS exposure

There are two main approaches to assess the additional medical spending attributable to smoking—inclusive and disease-specific.^18^ ^19^ The disease-specific approach attempts a priori to identify smoking-related diseases and their costs,^20^ or uses the absolute death rates from lung cancer as an indicator of tobacco-related deaths.^21^ The inclusive approach developed here recognises that the health effects of smoking are complex and multiple, and assesses the impact of smoking on all medical spending. This latter does not require accurate disease diagnosis (which may not be available in rural parts of China).

Crude and age-standardised analyses demonstrated higher health service use and expenditure among those who had quit smoking, but not among current smokers, from the NHSS data. To examine the effect of smoking, the following regression equation of medical care utilisation is estimated using a log-linear approach^19^:


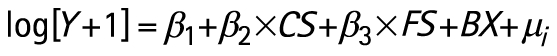


where *Y* is the annual self-reported medical expenditure of a household, which might be zero (hence the need to add 1 before logging); CS and FS are dummy variables equal to 1 if the individual is a current smoker (CS) or former smoker (FS); and X is the vector of demographic and individual covariates with coefficient vector *B*.

For ETS, a similar regression equation is estimated:


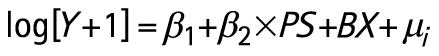


where PS is a dummy variable, equal to 1 if the individual lives in a household in which there is at least one current smoker, and no “smoke-free” household policy; and X is the vector of the same demographic and individual covariates as before with coefficient vector *B*. We have therefore conservatively assumed that there are no medical costs relating to ETS among households with former smokers.

We used the log-linear model to control for respondents’ age, gender, marital status, level of education, occupation, health insurance status (yes, no) and drinking status (see appendices 2–4). Household level variables we controlled for included household location (urban or rural area), household income and the distance from respondent’s home to the nearest medical institution.

In both of the models, our reference category is households that contained only never-smokers. Additionally, as the NHSS survey only collected data on smoking status from those aged 15 and above, we only included individuals over 15 in the reference category when estimating the medical care expenditures associated with smoking.

We then predicted the value of the dependent variable for each current and former smoker using the coefficient estimates from the regression equations. To estimate the increase in medical expenditure associated with smoking, we used the same coefficient estimates but set the CS and FS variables to zero. Then, the predicted expenditure of the smokers (assuming they had never smoked) is subtracted from the predicted expenditure. The difference of these two expressions is the predicted effect of smoking on medical care expenditure.

## RESULTS

### Association between tobacco consumption and other household expenditure

Table 2 shows crude expenditure on different categories (yuan and %) by smoking status, showing that households with quitters spent more on medical care, and households with smokers less on education. We estimated the marginal propensity to spend on other expenditure categories in relation to tobacco consumption in all surveyed households using the fractional logit model. Tobacco consumption was negatively and significantly related to spending on education, and medical care (table 3). Consuming every five packs of cigarettes (100 cigarettes in total) per capita per month was associated with 16.6 (5% less) yuan, and 10.9 yuan declines (4% less) in the above consumption categories, respectively.

**Table 3:** The marginal effects of tobacco consumption on other household expenditures in the total survey population, and the poorest 20% of rural households in the survey

	β	Marginal effects			SE	p Value
		% Total income	Money value (Per capita per year (yuan))	Percentage of total expenditure on category		
**A Total population**						
Food	0.0074	0.18	5.89	0.6	0.0001	<0.0001
Clothing	−0.0010	−0.01	−0.29	−0.1	0.0002	<0.0001
Transport	0.0426	0.32	10.48	5.0	0.0002	<0.0001
Housing	0.0000	0.00	0.00	0.0	0.0002	00.9494
Education	−0.0438	−0.50	−16.60	−4.8	0.0002	<0.0001
Medical care	−0.0335	−0.33	−10.93	−3.8	0.0002	<0.0001
Others	0.0302	0.22	7.33	3.5	0.0001	<0.0001
**B Poorest 20% of rural households**						
Food	0.0126	0.29	2.74	0.8	0.0005	<0.0001
Clothing	0.0035	0.03	0.30	0.3	0.0009	0.0003
Transport	0.0645	0.30	2.76	6.1	0.0012	<0.0001
Housing	0.0160	0.12	1.07	1.5	0.0009	<0.0001
Education	−0.0447	−0.54	−5.05	−3.8	0.0009	<0.0001
Medical care	−0.0478	−0.68	−6.37	−3.9	0.0007	<0.0001
Others	0.0506	0.38	3.49	4.6	0.0009	<0.0001

The marginal effects of tobacco spending (per 1000 yuan) on other household expenditures in (a) total dataset (n = 56 839 households and 193 137 individuals; 77 missing households), and (b) poorest 20% households in rural China in 2003 (n = 10 611 households; 42 238 individuals; 36 missing households).

The unit of amount of cigarette consumption is five packs per capita per month (100 cigarettes, approximately three per day) in a household.

In smoking households, the actual reduction may be much higher as the average household with one male smoker consumed around 450 cigarettes per month in our survey.^22^ The results also indicated that tobacco consumption was positively and significantly associated with increased spending on transportation, food and spending in the “other” category, but the percentage changes were very small (<1%, table 3). Costs of tobacco are included in the “other” category, so spending on this category increased with higher tobacco consumption. In fact, the only variable not associated with tobacco consumption is housing, presumably because these generally are fixed costs for households.

### Association between tobacco consumption and other household expenditure among the poorest 20% of rural households

We repeated our analyses limiting the dataset to the poorest 20% of rural households. Tobacco consumption was associated with 5.1 yuan (4%) and 6.4 yuan (4%) declines in spending on education and medical care respectively (table 3). The average income of the poorest 20% of rural households was only 709 yuan per capita per year, so though absolute monetary values are small, the negative effect on households was quite severe.

### Medical spending associated with cigarette smoking

#### Active cigarette smoking

Appendices 1–3 (see website) display crude data and results of regression models quantifying associations between household smoking and medical expenditure. Medical expenditure is 2.7% higher (p = 0.04) among current smokers compared with never-smokers, and 27.0% higher among former smokers (p<0.0001) (table 4 and appendix 2 (see website)). Households with former smokers experience the greatest increase in medical costs, especially those with two or more former smokers (table 4), an additional 64.0 yuan per capita per year (84.9 in urban areas; 51.6 in rural areas).

**Table 4:** Excessive medical spending attributable to active smoking and exposure to environmental tobacco smoke (ETS) by smoking status (yuan, per capita per year)

Smoking status	National	Urban	Rural
**Active smoking**			
NS (reference category)	0.0	0.0	0.0
Low	3.1	4.1	2.7
Medium	3.2	4.0	2.9
High	4.2	5.2	4.0
1 quit	39.2	54.5	32.9
2+ quit	64.0	84.9	51.6
Mixed	34.9	45.1	30.6
**ETS**			
Low	13.5	14.1	13.3
Medium	13.8	14.7	13.6
High	11.9	12.2	11.8

NS, household of never-smokers; Low, low tobacco consumption (0–25% of distribution of tobacco consumption); Medium (25–75%) and high tobacco consumption (75–100%).

#### Environmental tobacco smoke (ETS)

We also estimated medical spending as a result of ETS using the same method, except that we included the whole population in the reference category (households with never-smokers), regardless of age.

Appendix 3 (see website) shows that among households with exposure to ETS there is a statistically significant (p<0.0001) 7% higher medical expenditure among non-smoking household members, compared with households with no ETS exposure. For low-tobacco consumption households, the average excessive medical spending attributable to ETS is 13.5 yuan per year (with similar results for moderate and high consumption households, table 4).

## DISCUSSION

Tobacco consumption drives out spending on basic household commodities, especially education, and on medical care. Our results reinforce those published from other Chinese surveys. A 2002 rural health insurance survey in two poor rural provinces in China showed that rural households spent 6.5% of their total expenditure on tobacco, and showed that for every 100 yuan spent on tobacco, there was a 30 yuan decrease in spending on education and a 15 yuan decrease on medical care.^9^ ^23^ A further study found that poor urban households spent 6.6% of their expenditure on cigarettes, and 11.3% in poor rural areas. Again, tobacco spending was associated with reductions in spending on other goods and services.^24^ This finding is a particular concern as expenditures on education and medical care both tend to increase productivity—that is, are human capital investments. As education expenditures mainly benefit children (from school fees), it is conceivable that children in poorer smoking households may be forced to give up some education. This has particular concerns for China’s continued economic and social development. Tobacco consumption was associated with higher food costs, but the food category included the costs of alcohol, and smokers are far more likely to drink alcohol regularly than non-smokers (crude OR = 8.99, 95% CI 8.63 to 9.37), which may account for this. Tobacco consumption was also associated with slightly increased spending on transport; it is possible that this is because of the association of smoking with social activities in China.^25^

There are two main conclusions from our analyses of medical expenditure by smoking status. One is that households with former smokers have very high medical spending, and the second is that ETS exposure is also associated with raised medical care expenditures. These costs are proportionally greater for the poorest 20% of rural households, where excessive medical spending due to smoking as a percentage of income is 3.5 times that for the highest income quintile.

Fractional logit modelling estimating the “direct” costs of smoking indicated that increases in tobacco consumption were associated with reductions in spending on medical care as a percentage of total household expenditure, while log-linear models estimating the “indirect medical care” costs associated with tobacco found that households with smokers had slightly increased absolute medical care costs. Although we controlled for household income within quintiles in the log-linear models, there is likely to be some residual confounding as even within the poorest quintile (and decile) of rural households, smoking households still had slightly higher incomes than non-smoking households, allowing for differences in percentage and absolute tobacco expenditure to arise. Particularly in rural areas, it is likely that those in the very poorest households do not smoke because of the economic burden. The appropriate interpretation is therefore that tobacco consumption reduces medical expenditure, but those in slightly wealthier households (even among poorer rural areas) are more likely to smoke, and also spend more on medical care, probably because of their slightly higher incomes.

It may seem surprising that the medical care costs of current smoking households are not higher, although the costs among households with former smokers were very high. Only direct household costs were collected by the survey. Smokers with health insurance, and in some cases their families, may be partly sheltered from the direct costs of excessive medical expenditure due to smoking-related diseases. This is unlikely to be a major factor though, as health insurance coverage was low in 2003 (nearly half of the urban population had no coverage at all),^13^ and out-of-pocket payments represent a high proportion of total healthcare expenditure in China (59% in 2000); in particular outpatient services (including primary care) are not covered at all in most parts of China.^26^ Probably more importantly, health service utilisation data do not necessarily reflect health need, and there is clear evidence from the NHSS that smoking households are less likely to obtain secondary medical care, even when this has been recommended by a doctor (data not shown). Finally, the survey was cross-sectional in design; smokers who become ill may decide to quit, resulting in a “healthy smoker” bias.^22^

Our data source was a very large, nationally representative household survey, the NHSS. We believe this is one of the first attempts to estimate the household-associated costs of smoking from a nationally representative survey in a developing world country. However, our analyses have several limitations. The main limitation is that the NHSS survey did not collect data on spending on cigarettes directly; we therefore used household tobacco consumption as the dependent variable. Cigarette prices vary considerably in China, and it has been reported elsewhere that while poor rural households overwhelmingly choose cheap local brands, richer urban households may purchase more expensive brands. We could have estimated cigarette price using data from other Chinese surveys,^24^ ^27^ but we preferred to use the tobacco consumption data, collected by the NHSS. Critically, we found the expected dose-response relation in both urban and rural areas—increasing categories of tobacco consumption reduced spending further on other categories, such as education and medical care. However, it is possible that our approach may underestimate the effects of smoking costs on wealthier urban households, if these are purchasing more expensive cigarette brands. We may also underestimate the direct costs of smoking through misclassification of smoking costs—as some households, categorised as “low” consumption may be buying more expensive cigarettes, while some categorised as “high” consumption may be buying cheap cigarettes. Most of our survey data (on cigarette consumption, household incomes and medical care costs) were self-reported, and thus may be subject to bias. However, we used trained local interviewers, and were able to validate some income data against household benefits and expenditures. Finally, our data are cross-sectional; even if smokers quit we do not know how households would use potential savings.

**What this paper adds**

- Households with current smokers spend significantly less on other goods and services, particularly education and medical care.
- Household medical care expenditure costs are substantially higher among households with one or more ex-smoker.
- Exposure to environmental tobacco smoke is also associated with increased medical care costs.
- The effects are most acute among poorer rural households, contribute to impoverishment and may have a substantial effect on China’s continued economic and social development.

Reducing smoking is not only critical in improving health, it also has an important role in poverty reduction in China, particularly in poorer rural areas. As well as the “direct” opportunity costs of smoking, reducing expenditure on education, the cost of medical care has been increasing rapidly in China over recent years compared with income, and the average cost of a single hospital admission is over double the average annual income of the poorest 20% of the population.^26^ It is therefore likely that the household medical costs associated with adult smoking have increased since the NHSS was carried out in 2003; and even if smoking falls, in absolute terms medical costs as a result of smoking are likely to rise further. It has been estimated that 35% of urban households and 43% of rural households have difficulty affording healthcare, go without healthcare, or are impoverished by health costs.^28^ Insurance coverage for most of the Chinese population is also inadequate.^26^ We found particularly high medical care costs among households with quitters; the true costs are probably far higher as many poorer smokers may be foregoing the healthcare they need.^22^

Policy initiatives should focus particularly on rural areas in China, where smoking prevalence and poverty are higher, and where sustained anti-smoking campaigns have been rare. Information on the opportunity costs of smoking should be widely disseminated in these areas. Smoking cessation advice and nicotine replacement therapies are not generally available in rural areas; their use should be promoted by doctors and covered by health insurance schemes. Extending and enforcing smoke-free policies in public places^13^ ^29^ and, more controversially, increasing taxes, have both been shown to reduce smoking prevalence in similar areas, and may have both economic and health benefits for China.^4^ ^30^ In China, assuming a price elasticity of −0.54, it has been estimated that a 40% increase in tax from 1.60 yuan per pack to 2.00 yuan tax per pack would reduce consumption by 4.57 billion packs, generate additional central government revenues of nearly 25 billion yuan, and save 1.44–2.16 million lives.^30^ The increase in central government tax revenue would be twice as large as the total losses to industry and agriculture.^16^ Our study suggests it would also reduce poverty and increase resources available for household spending on education and medical care, important for China’s future development.
